# Eruptive Syringomas on the Neck: Clinicopathological and Dermoscopic Features

**DOI:** 10.5826/dpc.1102a22

**Published:** 2021-03-08

**Authors:** Gargi Taneja, Neirita Hazarika, Riti Bhatia

**Affiliations:** 1 Department of Dermatology and Venereology, All India Institute of Medical Sciences, Rishikesh, India

**Keywords:** syringomas, eccrine gland neoplasms, neck, eruptive

## Introduction

Syringoma is a benign adnexal tumor that originates from the acrosyringium. Eruptive syringoma is a rare variant that manifests in the form of skin-colored or brownish, shiny, angulated papules that occur in successive crops. Dermoscopic evaluation reveals brownish regular pigment network and tiny whitish dots between adjacent papules that correspond histologically to multiple eccrine ducts lined by a double-layered epithelium, giving a paisley tie appearance. This case of eruptive syringomas presenting with brownish papules on the neck highlights the importance of histopathological and dermoscopic evaluation.

## Case Presentation

**Figure 1:**
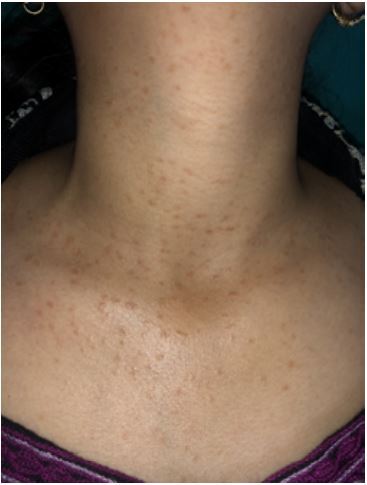
Multiple monomorphic brownish papules on the neck and upper chest.

A woman in her late twenties presented with a 7- to 8-year history of asymptomatic papules on the neck. The papules developed episodically, in clusters (Figure 1). She had no history of chronic disease and no family history of a similar condition. Physical examination revealed multiple monomorphic brownish papules on the anterior part of the neck and chest. Routine laboratory tests, including lipid profile and serum thyroid hormone levels, were all normal.

**Figure 2:**
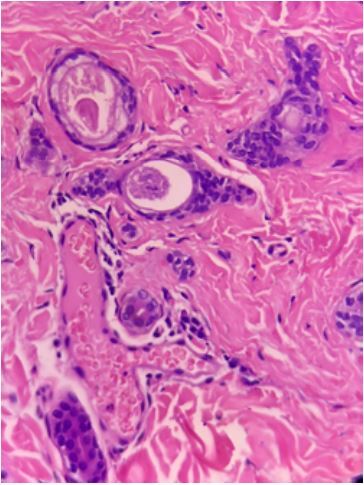
Lesional biopsy shows multiple eccrine ducts and solid nests of epithelial proliferation with comma-like structures, resembling a paisley tie pattern (H&E, ×400).

A 4-mm punch biopsy was obtained from one of the papules. Histopathologic analysis revealed multiple eccrine ducts and solid nests of epithelial proliferation within the dermis. The ducts were lined by a double-layered epithelium. Amorphous eosinophilic material could be appreciated in some ductal lumina. Some ducts showed small, comma-like extension of epithelial cells. The stroma was collagenous (Figure 2).

**Figure 3:**
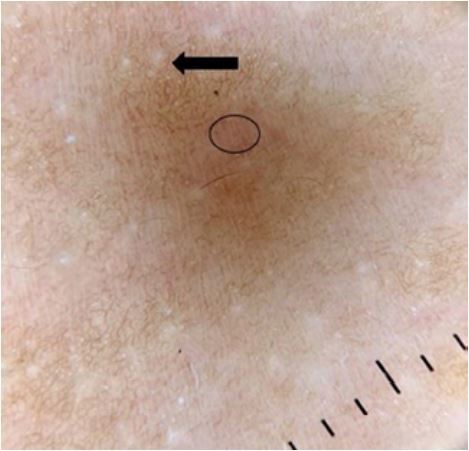
Dermoscopic image (polarized mode, DermLite) of the papule shows regular brownish pigmented network (circle) and multiple tiny white dots (black arrow).

Dermoscopic evaluation (polarized mode, DermLite) of a papule revealed brownish regular pigment network and tiny whitish dots between adjacent papules (Figure 3). Based on clinical, histopathogical, and dermoscopic features, a diagnosis of eruptive syringomas was made.

## Conclusions

Syringoma is a benign adnexal tumor that originates from the acrosyringium [1]. The commonest type of syringoma is the localized variant that usually involves the periorbital area. Eruptive syringoma is a rare variant that manifests around puberty in the form of skin-colored or brownish, shiny, angulated papules that occur in successive crops. Other variants include the familial type and trisomy 21-associated syringoma. Eruptive syringoma usually involves the anterior chest, upper abdomen, axillae, and periumbilical region [1]. It has been reported in association with Down syndrome, prurigo nodularis, sarcoidosis, psychiatric disorders, and Ehlers–Danlos syndrome. Clinical differential diagnoses include steatocystoma multiplex, disseminated xanthoma, multiple trichoepitheliomas, urticaria pigmentosa, and disseminated granuloma annulare.

Histopathological findings include eccrine ductal proliferation in a dense fibrous stroma. There is formation of nests and cords of double-layered epithelial cells. The outer epithelium is often elongated into comma-like structures, resembling a paisley tie pattern. Dermoscopic features include a delicate pigment network and multifocal whitish areas [2]. The pigment network has been attributed to overlying basal hyperpigmentation, more so in the eruptive variant. Whitish dots in between the pigment network corresponds to the opening of eccrine glands, which are larger than those of uninvolved skin [2]. However, dermoscopic features of syringoma are described somewhat variably in the literature. The reticular brown pigmentation may not to be present in all cases, especially in isolated syringoma [3]. Most cases of eruptive syringomas show a fine pigment network with whitish areas, but data is limited due to the sparse literature on this topic [4]. The rosette pattern of whitish dots has also been described in 1 case report [4].

The lesions are benign and usually remain static. Treatment modalities include dermabrasion, excision, electrodesiccation, chemical peeling, and oral and topical retinoids. The outcome is usually unsatisfactory. This case of eruptive syringomas presenting with brownish papules on the neck highlights the importance of histopathological and dermoscopic evaluation.

## References

[1] Pruzan DL, Esterly NB, Prose NS (1989). Eruptive syringoma. Arch Dermatol.

[2] Ankad BS, Sakhare PS, Prabhu MH (2017). Dermoscopy of non-melanocytic and pink tumors in brown skin: a descriptive study. Indian J Dermatopathol Diagn Dermatol.

[3] Corazza M, Borghi A, Minghetti S, Ferron P, Virgili A (2017). Dermoscopy of isolated syringoma of the vulva. J Am Acad Dermatol.

[4] Zhong P, Tan C (2015). Dermoscopic features of eruptive milium-like syringoma. Eur J Dermatol.

